# The Impact of Mental Health Conditions on Public Insurance Costs of Treating HIV/AIDS

**DOI:** 10.1007/s10461-019-02663-w

**Published:** 2019-09-06

**Authors:** Arleen A. Leibowitz, Katherine A. Desmond

**Affiliations:** Department of Public Policy, UCLA Luskin School of Public Affairs, 3250 Public Affairs Building, Box 951656, Los Angeles, CA 90095-1656 USA

**Keywords:** HIV/AIDS, Mental health, Treatment costs, Medicare, Medicaid managed care capitations

## Abstract

Medicare and Medicaid insurance claims data for Californians living with HIV are analyzed in order to determine: (1)The prevalence of treatment for particular mental health diagnoses among people living with HIV (PLWH) with Medicare or Medicaid insurance in 2010; (2)The relationship between individual mental health conditions and total medical care expenditures; (3)The impact of individual mental health diagnoses on the cost of treating non-mental health conditions; and (4)The implications of the cost of mental health diagnoses for setting managed care capitation payments. We find that the prevalence of mental health conditions among PLWH is high (23% among Medicare and 28% among Medicaid enrollees). PLWH with mental health conditions have significantly higher treatment costs for both mental health and non-mental health conditions. Setting managed care capitations that account for these greater expenditures is necessary to preserve access to both mental health and physical health services for PLWH and mental health conditions.

**Electronic supplementary material:**

The online version of this article (10.1007/s10461-019-02663-w) contains supplementary material, which is available to authorized users.

## Resumen

Datos de reclamos de seguro de Medicare y Medicaid para los Californianos que viven con el VIH se analizan para determinar: (1) La prevalencia de tratamiento de ciertos diagnósticos de salud mental entre las personas que viven con el VIH (PLWH por sus siglas en Inglés) con seguro de Medicare o Medicaid en 2010; (2) La relación entre las condiciones individuales de salud mental y los gastos totales de atención médica; (3) El impacto de los diagnósticos individuales de salud mental en el costo del tratamiento de afecciones no mentales; y (4) Las implicaciones del costo de los diagnósticos de salud mental para establecer pagos de capitación de atención administrada. Encontramos que la prevalencia de afecciones de salud mental entre las PLWH es alta (23% entre Medicare y 28% entre los afiliados a Medicaid). Las PLWH con afecciones de salud mental tienen costos de tratamiento significativamente más altos para las afecciones de salud mental y no mental. Es necesario establecer capitulaciones de atención administrada que tengan en cuenta estos gastos mayores para preservar el acceso tanto a los servicios de salud mental como a los de salud física para las PLWH y las condiciones de salud mental.

## Introduction

The prevalence of mental illness among people living with HIV (PLWH) is high [1–7]. Substantial percentages of PLWH have mood disorders [1, 2, 5, 8]. Rates of depression are three times as high among PLWH as among those who are seronegative [4, 8, 9]. In a meta-analysis, the pooled prevalence of depression across nine studies covering 7375 PLWH was 41% [10]. Even rates calculated over short time periods are substantial. The Medical Monitoring Project, a nationally representative sample of adults receiving care for HIV in the U.S., found that 25.6% had experienced depression in the prior two weeks, with 12.4% of the sample experiencing major depression [4, 8]. Similarly, Bengtson et al. found that 26% of HIV positive men and 29% of HIV positive women showed signs of depression [11]. In a diverse group of HIV-positive patients engaged in routine care, 39–44% showed signs of depression [12]. Similarly 28% of women with HIV and 19% of men with HIV were assessed with current depression [13]. Higher rates have been found in some subpopulations, especially HIV-positive minority men. In the mStudy, 65.2% of the primarily minority PLWH had a history of psychiatric illness and 56.8% were currently depressed [5]. Additionally, among HIV patients, 16.2% reported anxiety, a rate significantly higher than among HIV-negative patients (6.9%; p = .009) [ [7].]

Depressed PLWH are more likely to engage in high risk sexual behaviors that increase the chance of transmitting the HIV virus [14]. PLWH who have untreated mental health conditions also experience negative health outcomes due to lower rates of initiating and adhering to anti-retroviral therapy (ART) [10, 15, 16], but there were few significant relationships between anxiety disorders and adherence to ART. [17] Current depression is also linked to increased use of emergency departments and higher costs. [18] In a large urban treatment program in 2003, PLWH who were comorbid for Serious Mental Illness (SMI) had 85% greater Medicaid costs than persons with only HIV and twice the costs of persons with only a SMI diagnosis [19].

To better understand the added needs and public insurance treatment costs for PLWH who also have mental health issues, this study examines Medicaid and Medicare insurance claims data prior to the time when most PLWH in California covered by Medi-Cal (California’s Medicaid program) were moved into managed care. This paper provides a broad view of the impact of a number of mental health conditions on health care utilization by analyzing insurance claims data for Californians living with HIV who are covered by Medicare and Medicaid in order to determine:

1. The prevalence of treatment for particular mental health diagnoses among PLWH with Medicare and Medicaid insurance in California in 2010
2. The relationship between individual mental health conditions and total medical care expenditures
3. The impact of individual mental health diagnoses on the cost of treating non-mental health conditions
4. The implications of the cost of mental health diagnoses for setting managed care (MCO) capitations.

## Methods

### Data

Data for this analysis consist of Medicare and Medicaid insurance claims for Californians in 2010 acquired through a confidential data use agreement with the Centers for Medicare and Medicaid Services (CMS). We applied a case-identification algorithm to create an analysis file of adult beneficiaries with verifiable HIV [20]. Our analyses were limited to individuals enrolled for the full year in fee-for-service arrangements because available data for managed care enrollees lack diagnosis fields needed to confirm HIV status. Data acquisition was reviewed and approved by the CMS Privacy Board. Research was also approved for expedited review by the relevant Institutional Review Board (IRB #10-000823). Data were obtained in research identifiable files; storage, analysis, and reporting met CMS data security requirements.

### Individual Characteristics

#### Insurance

Medicare reimbursement rates generally exceed those in Medicaid, therefore we distinguish the two types of public insurance: The Medicare group includes aged and long-term disabled beneficiaries with HIV for whom Medicare is the primary payer. This group includes Dual eligibles who have Medicaid as a secondary payer. The Medicaid-Only group has Medicaid coverage, but no Medicare coverage.

#### Mental Health

Mental health disorders were classified using the Clinical Classifications Software (CCS) from the Health Care Utilization Project [21]. Diagnoses were identified by relevant ICD-9 diagnosis codes appearing in an individual’s inpatient medical claims at least once or at least twice in outpatient claims on different days. We grouped the CCS mental health diagnoses into four major categories: mood disorders, adjustment and anxiety disorders (combining two CCS diagnostic groups), schizophrenia and other psychoses, and all other mental health disorders (attention-deficit, conduct, and disruptive behavior disorders; delirium, dementia, amnestic, and other cognitive disorders; developmental disorders; disorders usually diagnosed in infancy, childhood, or adolescence; impulse control disorders; personality disorders; screening and history of mental health disorders; and miscellaneous mental health disorders). None of the diagnoses combined into the ‘other’ category had a prevalence rate greater than 1.5%. Information on alcohol and substance use disorders and suicidality were redacted by CMS. Patients could have more than one mental health diagnosis.

#### Comorbidities

The Charlson Comorbidity Index (CCI) is a validated method of classifying comorbidity documented in medical records to predict long- and short-term mortality [22]. We identified 16 comorbidities that are part of the Charlson scale (requiring a single inpatient claim or multiple outpatient claims on different days with relevant ICD-9 codes): myocardial infarction, congestive heart failure, peripheral vascular disease, cerebrovascular disease, dementia, chronic pulmonary disease rheumatic disease, peptic ulcer disease, mild liver disease, diabetes with chronic complications, diabetes without chronic complications, hemiplegia or paraplegia, renal disease, any malignancy, moderate or severe liver disease, or metastatic solid tumor. AIDS-defining conditions and substance use were not included as comorbidities. We classified individuals as having zero, one, or two or more Charlson comorbidities.

#### Other

CMS enrollment data provided information on participant age, race, and gender.

### Outcome Variables

Medical care expenditures were extracted from inpatient, outpatient, prescription drug, and long term care files (the latter limited to Medicaid-Only and dual Medicare-Medicaid enrollees). Expenditures included all costs reimbursed by Medicare and Medicaid plus costs paid by the patient (e.g. deductible and coinsurance). We disaggregated inpatient and outpatient expenditures into two categories: mental health expenditures were calculated by summing over claims that had a mental health diagnosis in the first diagnosis field; non-mental health expenditures came from claims with other diagnosis codes in the first field. We also calculated spending on antiretroviral (ARV) drugs, spending on other pharmaceuticals, and Medicaid expenditures for long term care. Drug and long term care expenditures were not broken out by whether they were for mental health.

### Statistical Analysis

All analyses were performed separately for Medicare and Medicaid-Only enrollees. Prevalence rates of mental health disorders were examined overall, and by individual characteristics. Chi square tests were used to identify prevalence rates that differed significantly by individual characteristics. We calculated mean outpatient expenditures, prevalence of inpatient stays, mean inpatient expenditures conditional on having an inpatient stay, mean expenditures on antiretroviral medications and other prescription medications, and on long term care—overall and by mental health diagnosis. Outpatient and inpatient spending was also broken out by whether it related to mental health or non-mental health care. *T* tests compared differences in mean spending for enrollees with and without any mental health diagnoses.

To control for multiple influences on health spending, we estimated multivariable regressions that related medical care spending to demographics, type of insurance, mental health diagnoses and other comorbidities. Outpatient spending was examined in one step since all the members of the analysis file had outpatient utilization. The cost of inpatient use was estimated in two steps: a logistic regression examined the factors related to hospitalization; a multivariable regression then examined inpatient expenditures conditional on any hospital use. Due to the skewness of the data, we transformed each individual’s outpatient and conditional inpatient expenditure with a natural logarithm. We present estimated percentage changes and odds ratios showing the impact of the mental health variables in each regression, controlling for demographic and health variables. Full results from each model are provided in an Online Appendix.

## Results

Mental health diagnoses were frequent in our analysis file of 17,000 Californians with HIV enrolled in Medicare and/or Medicaid. About ¼ of enrollees were treated for a mental health diagnosis in 2010 (23.0% of those with any Medicare coverage and 28.1% of Medicaid-only enrollees) (Table 1). The most prevalent diagnosis was mood disorders (16–18%). However, adjustment/anxiety disorders (5.6–6.2%) were not uncommon and there was a high prevalence of psychoses, including schizophrenia, particularly among the PLWH with only Medicaid insurance (8.1%). Four percent of persons with Medicare had received a diagnosis of psychosis. Nearly one quarter (23.1%) of the group had at least one of these three diagnoses, with 3.1–4.8% having any of the other mental health diagnoses.

**Table 1:** Mental health diagnosis by demographic characteristics and type of public insurance

	Total population	Any mental health diagnosis	P* (χ^2^)	Mood disorder	P* (χ^2^)	Adjustment/anxiety	P* (χ^2^)	Psychoses	P* (χ^2^)	Other mental health diagnosis	P* (χ^2^)
	N, % of population in row	N, % of row with diagnosis									
Medicare incl. dual	11,355 (100%)	2609 (23.0%)		1841 (16.2%)		638 (5.6%)		457 (4.0%)		354 (3.1%)	
Gender			.005 (8.0)		.742 (0.11)		< .001 (18.0)		.003 (9.0)		< .001 (13.9)
Male	10,146 (89.4%)	2292 (22.6%)		1641 (16.2%)		538 (5.3%)		389 (3.8%)		295 (2.9%)	
Female	1209 (10.7%)	317 (26.2%)		200 (16.5%)		100 (8.3%)		68 (5.6%)		59 (4.9%)	
Race/ethnicity			< .001 (36.0)		< .001 (42.7)		<.001 (19.0)		< .001 (26.9)		.047 (6.1)
White	6611 (58.2%)	1648 (24.9%)		1197 (18.1%)		418 (6.3%)		230 (3.5%)		225 (3.4%)	
African-American	2094 (18.4%)	437 (20.9%)		294 (14.0%)		81 (3.9%)		125 (6.0%)		65 (3.1%)	
Hispanic	2154 (19.0%)	435 (20.2%)		280 (13.0%)		112 (5.2%)		87 (4.0%)		64^a^ (2.4%)	
Other	496 (4.4%)	89 (17.9%)		70 (14.1%)		27 (5.4%)		15 (3.0%)			
Age			< .001 (86.1)		< .001 (92.8)		< .001 (17.9)		< .001 (73.5)		.099 (4.6)
18–34	342 (3.0%)	89 (26.0%)		60 (17.5%)		23 (6.7%)		30 (8.8%)			
35–49	4319 (38.0%)	1089 (25.2%)		778 (18.0%)		271 (6.3%)		234 (5.4%)		138^b^ (3.0%)	
50–64	4942 (43.5%)	1176 (23.8%)		855 (17.3%)		281 (5.7%)		164 (3.3%)		147 (3.0%)	
65 or older	1752 (15.4%)	255 (14.6%)		148 (8.4%)		63 (3.6%)		29 (1.7%)		69 (3.9%)	
Enrollment			< .001 (82.2)		< .001 (58.8)		< .001 (16.3)		<.001 (76.0)		.089 (2.9)
Medicare only	3748 (33.0%)	670 (17.9%)		466 (12.4%)		164 (4.4%)		65 (1.7%)		102 (2.7%)	
Dual Medi-Medi	7607 (67.0%)	1939 (25.5%)		1,375 (18.1%)		474 (6.2%)		392 (5.2%)		252 (3.3%)	
Charlson conditions			< .001 (59.7)		<< 001 (36.7)		< .001 (28.6)		< .001 (40.1)		< .001 (96.0)
No comorbidities	6854 (60.4%)	1413 (20.6%)		998 (14.6%)		324 (4.7%)		220 (3.2%)		140 (2.0%)	
One comorbidity	2912 (25.7%)	743 (25.5%)		529 (18.2%)		191 (6.6%)		133 (4.6%)		108 (3.7%)	
Two or more	1589 (14.0%)	453 (28.5%)		314 (19.8%)		123 (7.7%)		104 (6.5%)		106 (6.7%)	
Medicaid Only											
N	6245 (100%)	1755 (28.1%)		1,109 (17.8%)		385 (6.2%)		505 (8.1%)		300 (4.8%)	
Gender			.021 (5.4)		.096 (2.8)		.162 (2.0)		.962 (.002)		.055 (3.7)
Male	4508 (72.2%)	1230 (27.3%)		778 (17.3%)		266 (5.9%)		365 (8.1%)		202 (4.5%)	
Female	1737 (27.8%)	525 (30.2%)		331 (19.1%)		119 (6.9%)		140 (8.1%)		98 (5.6%)	
Race/ethnicity			.025 (9.3)		< .001 (30.1)		< .001 (20.7)		< .001 (27.1)		.057 (7.5)
White	2152 (34.5%)	655 (30.4%)		445 (20.7%)		172 (8.0%)		154 (7.2%)		113 (5.3%)	
African-American	2156 (34.5%)	570 (26.4%)		316 (14.7%)		102 (4.7%)		220 (10.2%)		112 (5.2%)	
Hispanic	1232 (19.7%)	335 (27.2%)		236 (19.2%)		70 (5.7%)		68 (5.5%)		41 (3.3%)	
Other	705 (11.3%)	195 (27.7%)		112 (15.9%)		41 (5.8%)		63 (8.9%)		34 (4.8%)	
Age			.135 (4.0)		.277 (2.6)		.032 (6.9)		.056 (5.8)		< .001 (27.0)
18-34	615 (9.9%)	186 (30.2%)		117 (19.0%)		49 (8.0%)		43 (7.0%)		54 (8.8%)	
35-49	2887 (46.2%)	831 (28.8%)		528 (18.3%)		188 (6.5%)		259 (9.0%)		141 (4.9%)	
50 or older	2743 (43.9%)	738 (26.9%)		464 (16.9%)		148 (5.4%)		203 (7.4%)		105 (3.8%)	
Charlson conditions			< .001 (34.2)		< .001 (18.6)		<.001 (21.5)		<.001 (51.5)		< .001 (30.3)
No comorbidities	4018 (64.3%)	1042 (25.9%)		673 (16.8%)		206 (5.1%)		254 (6.3%)		153 (3.8%)	
One comorbidity	1563 (25.0%)	473 (30.3%)		279 (17.9%)		122 (7.8%)		164 (10.5%)		92 (5.9%)	
Two or more	664 (10.6%)	240 (36.1%)		157 (23.6%)		57 (8.6%)		87 (13.1%)		55 (8.3%)	

^a^Hispanic and Other race/ethnicity combined to conform to CMS cell size restrictions

^b^34 or under and 35–49 combined to conform to CMS cell size restrictions

*P value and χ^2^ statistic from Chi square test of diagnosis rates, comparing row categories

Female Medicare recipients were more likely to have a mental health diagnosis (26.2%) than male Medicare recipients (22.6%) (χ^2^ = 8.0, p = .005) (Table 1). Among Medicaid-Only recipients, women were also more likely than men to be diagnosed with a mental health condition (30.2% of women vs. 27.3% of men) (χ^2^ = 5.4, p = .02). Women with Medicaid-Only were as likely as men (8.1%) to be diagnosed with a psychosis. Prevalence of mental health conditions differed by race/ethnicity in both the Medicare (χ^2^ = 36.0, p < .001) and Medicaid-Only (χ^2^ = 9.3, p = .025) groups, with Whites more likely than either African Americans or Hispanics to have a mental health diagnosis. Mental health diagnoses differed by age among Medicare recipients (χ^2^ = 86.1, p < .001), with a lower rate among those over 65 (14.6%) than among younger enrollees (23.8–26.0%). People with more comorbidities had significantly greater rates of mental health diagnosis in both the Medicare (χ^2^ = 59.7, p < .001) and Medicaid-Only group (χ^2^ = 34.2, p < .001). The prevalence of mental health disorders was significantly higher among the Medicare population with Dual coverage (25.5%), compared to those with Medicare only (17.9%) (χ^2^ = 82.2, p < .001).

Table 2 shows that mean health care expenditures for PLWH who have a mental health diagnosis are significantly higher than for those with no mental health diagnosis ($62,650 vs. $42,319 in Medicare, t = 14.8, p < .001; and $49,788 vs. $34,934 in Medicaid, t = 12.9, p < .001). Costs of mental health care contributed to this differential, averaging $4523 for Medicare recipients and $4787 for Medicaid-Only recipients with mental health diagnoses (15.2% of Medicare outpatient and inpatient expenses and 23.5% of comparable Medicaid expenses). Mental health costs are particularly high among patients with psychoses both in Medicare ($18,943) and Medicaid-Only ($10,213). Half of patients with psychosis in Medicare (53.6%) and nearly half of all Medicaid-Only enrollees with a mental health condition other than depression (48.3%) were hospitalized in 2010.

**Table 2:** Mean medical and mental health expenditures by mental health diagnosis and type of public insurance

	Total population	No mental health diagnosis	Any mental health diagnosis	*T* test of spending, any MH dx vs none P (t statistic)	Mood disorder	Adjustment/anxiety	Psychoses	Any other MH diagnosis
Medicare incl. dual								
N	11,355	8746	2609		1841	638	457	354
Total Expenditures	46,991	42,319	62,650	< .001 (14.8)	63,811	68,338	82,890	89,496
Outpatient	11,446	10,596	14,296	< .001 (7.1)	14,819	16,529	16,897	19,918
Inpatient (incl. $0)	7030	5091	13,531	< .001 (10.6)	13,758	17,010	28,522	27,822
ARVs	19,122	19,066	19,309	.309 (0.9)	19,589	19,314	17,681	17,263
Other Rx	7519	6504	10,919	< .001 (10.1)	11,705	10.974	10,582	10,786
Other, incl. LTC	1874	1062	4595	< .001 (10.8)	3940	15,474	9208	13,707
% With any inpt. $	19.3%	16.4%	29.2%	< .001* (213)	30.0%	30.3%	53.6%	40.7%
Inpt. cond. on > $0	36,418	31,137	46,329	< .001 (6.2)	45,886	56,229	53,202	68,396
Mental health	1045	8	4523	< .001 (11.6)	5488	7467	18,943	10,796
MH outpatient	268	8	1140	< .001 (20.3)	1395	1502	2859	1612
MH inpatient	777	0	3383	< .001 (9.4)	4093	5965	16,084	9184
Non-MH	17,431	15,679	23,304	< .001 (7.2)	23,089	26,072	26,476	36,944
Non-MH outpatient	11,178	10,588	13,156	< .001 (5.0)	13,424	15,027	14,038	18,306
Non-MH inpatient	6253	5091	10,148	< .001 (6.4)	9665	11,045	12,438	18,638
MH as % of outpt + inpt	3.6%	0.2%	15.2%	< .001 (20.7)	16.9%	16.8%	40.6%	17.1%
Medicaid only								
N	6245	4490	1755		1109	385	505	300
Total	39,108	34,934	49,788	< .001 (12.9)	49,610	55,383	57,097	67,922
Outpatient	8606	7208	12,181	< .001 (12.5)	11,875	14,782	15,068	19,216
Inpatient (incl. $0)	5307	4269	7963	< .001 (5.8)	8009	11,227	10,694	14,057
ARVs	16,771	16,802	16,691	.699 (0.4)	16,941	16,474	15,679	15,693
Other Rx	6544	5654	8822	< .001 (8.5)	9081	9304	9895	9831
Other incl. LTC	1880	1001	4131	< .001 (7.6)	3704	3596	5761	9125
% With any inpt. $	24.6%	20.4%	35.3%	< .001* (153)	37.0%	47.0%	48.1%	48.3%
Inpt. cond. on > $0	21,606	20,973	22,539	.392 (0.9)	21,664	23,881	22,225	29,083
Mental health	1355	13	4787	< .001 (14.9)	5037	8415	10,213	12,952
MH outpatient	1062	13	3746	< .001 (13.8)	3574	5203	6887	10,123
MH inpatient	293	0	1042	< .001 (8.2)	1463	3211	3326	2829
Non-MH	12,558	11,465	15,356	< .001 (5.0)	14,847	17,595	15,549	20,321
Non-MH outpatient	7544	7195	8435	< .001 (4.3)	8301	9578	8181	9093
Non-MH npatient	5014	4269	6921	< .001 (4.3)	6546	8016	7368	11,228
MH as % of outpt + inpt	6.8%	0.3%	23.5%	< .001 (35.8)	24.7%	27.8%	39.1%	34.7%

*P-value and test statistic from Chi square test

However, the greater treatment costs for mental health conditions do not fully account for the greater medical spending of PLWH with a mental health diagnosis compared to those without such a diagnosis. Table 2 shows that health care expenditures were higher in every category, including for non-MH conditions, for people with mental health diagnoses than for those without, with the exception of expenditures for ARVs and conditional inpatient Medicaid spending, where the two groups were very similar.

To estimate the net relationship between medical expenditures and mental health diagnoses, we estimated multivariable regressions that control for the differences in demographics and in comorbidities shown in Table 1 between persons with a mental health condition and those without such a diagnosis.

The regressions allow us to isolate the effect of each particular mental health condition on health expenditures, since the conditions were not defined to be mutually exclusive. Table 3 presents the estimated percentage increase in spending for total inpatient and outpatient care and for non-mental health inpatient and outpatient spending that is related to each mental health condition. Each of the mental health diagnoses was positively related to total outpatient costs, even after controlling for demographics, comorbidities, and other mental health conditions. On net, mood disorders were associated with more than 50% greater total costs for outpatient health care (52.9% in Medicare, t = 15.8, p < .001; 52.8% in Medicaid-Only, t = 12.2, p < .001). Medicare enrollees with an adjustment/anxiety diagnosis had 38.8% greater outpatient costs than Medicare enrollees without that diagnosis (t = 7.8, p < .001); Medicare enrollees with psychoses had 32.6% greater costs (t = 5.7, p < .001); and persons with other mental health diagnoses had 23% greater costs (t = 3.8, p < .001) compared to PLWH who did not have these diagnoses. For Medicaid-Only enrollees, each mental health diagnosis type was associated with more than 50% greater outpatient spending than PLWH without that mental health diagnosis (52.8% for mood disorders, t = 12.2, p < .001; 50.5% for Adjustment/Anxiety, t = 7.5, p < .001; 57.8% for Psychoses, t = 9.3, p < .001; 62.4% for other mental health diagnoses, t = 7.9, p < .001 (Table 3). Hospitalization rates were also significantly higher (except in the case of Medicare enrollees with adjustment/anxiety disorders) for beneficiaries with a mental health diagnosis (p < .01).

**Table 3:** Predicted net effect of mental health diagnosis on total and non-mental health spending for medicare and medicaid enrollees with HIV

Outcomes	Impact of having a diagnosis of:			
	Mood disorders	Adjustment/anxiety disorders	Psychoses	Other mental health disorders
Medicare				
All care				
% Increase in outpatient expenditures	52.9%***	38.8%***	32.6% ***	23.0% ***
Odds of inpatient stay	1.60 ***	1.13	3.87 ***	1.47 **
% Increase in inpatient expenditures^a^	− 3.3%	39.7% **	23.6% *	50.8% ***
Non-mental-health care				
% Increase in outpatient expenditures	38.5%***	31.5%***	15.8%**	21.1%***
Odds of inpatient stay	1.25**	1.13	1.435 **	1.43**
% Increase in inpatient expenditures^a^	0.1%	24.1%	14.6%	34.6%*
Medicaid				
All care				
% Increase in outpatient expenditures	52.8%***	50.5%***	57.8% ***	62.4%***
Odds of inpatient stay	1.65 ***	2.02***	2.09 ***	1.70***
% Increase in inpatient expenditures^a^	−14.8% *	9.8%	7.2%	34.6%**
Non-mental-health care				
% Increase in outpatient expenditures	22.4%***	29.6%***	− 1.1%	14.4%*
Odds of inpatient stay	1.24*	1.54***	1.08	1.35*
% Increase in inpatient expenditures^a^	− 11.5%	− 10.7%	− 1.9%	29.1%*

Estimates represent the net effect of each mental health diagnosis, based on regressions controlling for gender, race, age group, other MH diagnoses, number of comorbidities, and dual enrollment for Medicare. Outpatient and conditional inpatient expenditure analyses were based on logged expenditures (with exponentiated coefficients providing percent changes). Exponentiated logit estimates were used to estimate the odds of an inpatient stay. P-values are from *t* tests of zero effect for expenditures, Chi square tests of zero effect for odds of inpatient stay. Complete regression results are presented in the Online Appendix

*p < .05, **p < .01, ***p < .001

^a^Conditional on any inpatient stay

The cost of mental health treatment contributed to the higher total outpatient costs and greater use of inpatient services. Furthermore, PLWH who had mental health conditions also spent more on services that were not related to mental health. Spending on non-mental health outpatient care was 38.5% higher for Medicare enrollees with mood disorders (t = 12.1, p < .001), and 22.4% higher for Medicaid (t = 5.8, p < .001). Persons with adjustment/anxiety disorder spent approximately 30% more on outpatient medical care for non-mental health issues (31.5% more in Medicare, t = 6.5, p < .001; 29.6% more in Medicaid, t = 4.7, p < .001) (Table 3). Medicare recipients with psychoses spent 15.8% more on non-mental health outpatient care (t = 3.0, p < .01), but there was no significant increase among Medicaid-Only recipients with psychoses (t = − 0.2, p = 0.8171). PLWH with mental health diagnoses for the most part had greater odds of being hospitalized for a non-mental health condition (except for Medicare enrollees with adjustment/anxiety (χ^2^ = 1.3, p = .25) and Medicaid-Only enrollees with psychoses) (χ^2^ = .44, p = .50). However, once hospitalized, patients with mood disorders, adjustment/anxiety disorder or psychoses did not have greater inpatient spending than other inpatients without a mental health diagnoses.

## Discussion

This study of publicly insured Californians with HIV documented high levels of treatment for mental health conditions, particularly mood disorders. We find that about 1/6 of people in treatment for HIV received a diagnosis of mood disorders (16.2% of Medicare and 17.8% of Medicaid recipients.) These estimates, based on claims data, are comparable to those based on medical record and survey data. For example, 21.7% of a sample of PLWH receiving care in North Carolina and 25% of PLWH receiving care in Alabama were found to be depressed based on self-reports on the Patient Health Questionnaire (PHQ) [13, 23]. A study of HIV patients in London also fielded the PHQ and found that 19.8% of the patients were depressed [7]. Twenty-two percent of the nationally representative MMP sample reported depressive symptoms on the PHQ in a two-week period [24].

Anxiety/adjustment disorders are also prevalent among Medicare (5.6%) and Medicaid (6.2%) enrollees. These analyses found high prevalence of psychoses among Medicare enrollees (4.0%), and twice that rate among Medicaid enrollees (8.1%). These findings extend the existing literature, which focuses on mood disorders and relies on smaller clinical samples for other mental health conditions [25].

Medicare health care costs for PLWH who had a mental health diagnosis averaged $62,600 per year in 2010, compared to $42,300 for PLWH without a mental health diagnosis. Among the Medicaid-Only group the cost differential was $49,800 vs. $34,900. The effect sizes varied across diagnoses. Individuals with psychoses averaged annual costs of $82,900, which was primarily attributable to greater inpatient costs driven by high rates of hospitalization (53.6% in Medicare and 48.1% in Medicaid). Our multivariable analyses, which control for patient demographics and comorbidities, show that Medicare enrollees with a mental health diagnosis spent 23% to 53% more on outpatient care than those without a mental health diagnosis. Medicaid PLWH with mental health diagnoses had more than 50% greater outpatient costs than those without. Mental health conditions also had a dramatic effect on the odds of being hospitalized.

Our approach is innovative in examining not only the cost of mental health treatment, but also the impact of mental health conditions on non-mental health spending. On average, treatment costs for mental health conditions accounted for 15.2% of total annual inpatient and outpatient costs.

Not only did PLWH who had a mental health diagnosis incur costs to receive mental health care of $4000 to $5000 per year, but they also had greater costs for non-mental health care. In particular, individuals with both mood disorders and adjustment/anxiety disorders had elevated expenditures on non-mental health care in the outpatient sector.

Untreated mental health conditions negatively impact HIV outcomes. A meta-analysis found that PLWH with depression were significantly more likely to not use ART [10]. However, we found that use of ARVs did not differ significantly between the Medicare and Medicaid enrollees with and without a mental health diagnosis. Depressed patients in the mSTUDY had lower rates of adherence to ART and were less likely to be virally suppressed [5]. In a sample of community-recruited impoverished PLWH who used ART, those who screened positive for severe mental illness had viral loads that were six times greater than for the group that did not show signs of severe mental illness [26]. A meta-analysis showed that poor mental health relates not only to greater levels of viral load and lower CD4 counts, but also to an increased risk of death [27, 28].

Mental health treatment can ameliorate these effects by improving retention in care. [23] Among ART users, mental health treatment has been shown to increase adherence to ART [26, 29]. HIV patients visiting clinics that provided psychiatric, psychologic and social services as well as treatment for Hepatitis are more than three times as likely to achieve viral suppression as patients receiving treatment in clinics that offered only HIV primary care [30].

Given the deleterious effects of untreated mental health conditions on viral load, mortality, and HIV transmissions, it is vital that public insurance programs provide adequate access to mental health treatment for PLWH. In addition to the advantages for the PLWH, the community benefits because PLWH who are virally suppressed are less likely to transmit the virus.

The US National HIV/AIDS Strategy emphasizes the need for interdisciplinary HIV care that incorporates behavioral health treatment, such as that provided by Ryan White providers. Since its inception, the Ryan White program has emphasized the need for interdisciplinary HIV care that incorporates behavioral health treatment. An unintended consequence of the Affordable Care Act’s Medicaid Expansion was that this comprehensive care package, including treatment for behavioral issues in addition to medical treatment, became less available to many low-income PLWH who had been receiving care at Ryan White sites prior to 2014. Many Ryan White patients were required to enroll in Medicaid since Ryan White is designed to serve the uninsured and underinsured. Most of the new Medicaid eligibles in California had to enroll in managed care organizations (MCOs), which are compensated with a fixed monthly capitation to provide all necessary treatment.

Setting MCO capitation rates that reflect expected treatment costs is essential to insuring access to both physical and mental health treatment for PLWH. The movement of most California Medicaid enrollees with HIV into MCOs increases the urgency of understanding how mental health conditions affect the need for services—both treatment for mental health conditions and non-mental health conditions. Although MCOs currently receive an enhanced capitation for both Dual eligibles and Medicaid-Only enrollees with AIDS, there is not an enhanced rate for HIV alone. ART costs are carved out of most Medicaid MCO capitation rates in California, thereby preserving access to critical antiretroviral therapy. Since 2014, services for more severe mental health problems have also been carved out of Medi-Cal managed care contracts, and are provided by the counties [31, 32]. However, this paper shows that simply carving out treatment for severe mental illness from the costs that managed care organizations are required to pay does not adequately compensate the MCOs for the added costs of PLWH who have mental health diagnoses, including for hospitalization and for treatment for conditions other than mental health.

### Limitations

The analysis uses data that relate to a single period in time (2010), thus it is not possible to determine causality. It is possible that being hospitalized led to depression rather than depression leading to hospitalization. However, the finding that most mental health conditions were also associated with more frequent outpatient and inpatient use for non-mental health conditions, provides support for the causal effect running from mental health conditions to utilization rather than vice versa. We chose to use data for 2010, a period when most of the Medicaid enrollees were treated in the fee-for-service system because it allows us to examine utilization for severe mental health conditions, which is not possible with more recent periods in which California counties assume responsibility for these costs.

Second, since claims were used to identify having a mental health condition, some PLWH with a mental health condition for which they did not receive care may not have been identified. In that case, the prevalence of mental health conditions would have been even greater than estimated here. Per capita costs of mental health care may consequently have been overestimated because some PLWH were not treated for their mental health condition. The effect of this omission on non-mental health spending cannot be predicted.

Finally, levels of Medicaid expenditures for PLWH may not be directly generalizable to all states because California has relatively low levels of Medicaid reimbursement, averaging only 66% of the national mean reimbursement for primary care services [33]. The low reimbursement rates may affect the absolute ldifference in spending by PLWH with and without mental health conditions. However, since Medi-Cal’s reimbursement rates are low for all services, the findings regarding the percentage increase in non-medical spending and total spending that are related to having a mental health condition should be robust to differences in reimbursement levels. This conclusion is supported by the finding of similar relative expenditure patterns among persons with and without mental health diagnosis covered by Medicare, which has a largely national fee schedule.

As a result of the low reimbursement rates, Medi-Cal recipients may have had difficulty accessing mental health services through their providers and sought mental health care from Ryan White sites. If this were the case, actual mental health care needs would be even greater than documented here.

## Conclusion

The prevalence of mental health conditions among PLWH covered by Medicare and Medicaid is high. The greater expenditures for those with mental health conditions are not only attributable to their use of mental health services, but also to higher treatment costs for non-mental health conditions. In order to preserve access to both mental health and physical health services it is important to account for this additional non-mental health use when setting capitations for PLWH within managed care systems.

## Electronic supplementary material

Below is the link to the electronic supplementary material.

Electronic supplementary material 1 (DOCX 27 kb)

## References

[1] CDC. Behavioral and Clinical Characteristics of Persons with Diagnosed HIV Infection. June 2019.

[2] Israelski DM, Prentiss DE, Lubega S, Balmas G, Garcia P, Muhammad M (2007). Psychiatric co-morbidity in vulnerable populations receiving primary care for HIV/AIDS. AIDS Care.

[3] Bing EG, Burnam MA, Longshore D, Fleishman JA, Sherbourne CD, London AS (2001). Psychiatric disorders and drug use among human immunodeficiency virus-infected adults in the United States. Arch Gen Psychiatry.

[4] Blair JM, McNaghten AD, Frazier EL, Skarbinski J, Huang P, Heffelfinger JD. Clinical and behavioral characteristics of adults receiving medical care for HIV infection—Medical Monitoring Project, United States, 2007. Morbidity and mortality weekly report Surveillance summaries (Washington, DC: 2002). 2011;60(11):1–20. 21881551

[5] Aralis HJ, Shoptaw S, Brookmeyer R, Ragsdale A, Bolan R, Gorbach PM (2018). Psychiatric illness, substance use, and viral suppression among hiv-positive men of color who have sex with men in Los Angeles. AIDS Behav.

[6] Fletcher JB, Reback CJ (2017). Mental health disorders among homeless, substance-dependent men who have sex with men. Drug Alcohol Rev.

[7] McGowan JA, Brown J, Lampe FC, Lipman M, Smith C, Rodger A (2018). Resilience and physical and mental well-being in adults with and without HIV. AIDS Behav.

[8] Do AN, Rosenberg ES, Sullivan PS, Beer L, Strine TW, Schulden JD (2014). Excess burden of depression among HIV-infected persons receiving medical care in the united states: data from the medical monitoring project and the behavioral risk factor surveillance system. PLoS ONE.

[9] Bing EGBM, Longshore D (2001). Psychiatric disorders and drug use among human immunodeficiency virus-infected adults in the United States. Arch Gen Psychiatry.

[10] Tao J, Vermund SH, Qian HZ (2018). Association between depression and antiretroviral therapy use among people living with HIV: a meta-analysis. AIDS Behav.

[11] Bengtson AM, Pence BW, Powers KA, Weaver MA, Mimiaga MJ, Gaynes BN (2018). Trajectories of depressive symptoms among a population of HIV-infected men and women in routine HIV care in the United States. AIDS Behav.

[12] Cholera R, Pence BW, Bengtson AM, Crane HM, Christopoulos K, Cole SR (2017). Mind the gap: gaps in antidepressant treatment, treatment adjustments, and outcomes among patients in routine HIV care in a multisite U.S. Clinical Cohort. PLoS ONE.

[13] Ogburn DF, Schoenbach VJ, Edmonds A, Pence BW, Powers KA, White BL (2019). Depression, ART adherence, and receipt of case management services by adults with HIV in North Carolina, medical monitoring project, 2009–2013. AIDS Behav.

[14] Card KG, Lachowsky NJ, Armstrong HL, Cui Z, Wang L, Sereda P (2018). The additive effects of depressive symptoms and polysubstance use on HIV risk among gay, bisexual, and other men who have sex with men. Addic Behav.

[15] Gonzalez JS, Batchelder AW, Psaros C, Safren SA (2011). Depression and HIV/AIDS treatment nonadherence: a review and meta-analysis (1999). J Acquir Immune Defic Syndr.

[16] Kacanek D, Jacobson DL, Spiegelman D, Wanke C, Isaac R, Wilson IB (2010). Incident depression symptoms are associated with poorer HAART adherence: a longitudinal analysis from the Nutrition for Healthy Living study (1999). J Acquir Immune Defic Syndr.

[17] Springer SA, Dushaj A, Azar MM (2012). The impact of DSM-IV mental disorders on adherence to combination antiretroviral therapy among adult persons living with HIV/AIDS: a systematic review. AIDS Behav.

[18] Choi SKY, Boyle E, Cairney J, Grootendorst P, Gardner S, Collins EJ (2018). Impact of depression and recreational drug use on emergency department encounters and hospital admissions among people living with HIV in Ontario: A secondary analysis using the OHTN cohort study. PLoS ONE.

[19] Rothbard AB, Miller K, Lee S, Blank M (2009). Revised cost estimates of medicaid recipients with serious mental illness and HIV-AIDS. Psychiatric services (Washington, DC)..

[20] Leibowitz AA, Desmond K (2015). Identifying a sample of HIV-positive beneficiaries from Medicaid claims data and estimating their treatment costs. Am J Public Health.

[21] Clinical Classifications Software (CCS) 2015. [Internet]. U.S. Agency for Health Care Research and Quality. www.hcup-us.ahrq.gov/toolssoftware/ccs/ccs.jsp (2015). Accessed 4 Jan 2018.

[22] Charlson ME, Charlson RE, Peterson JC, Marinopoulos SS, Briggs WM, Hollenberg JP (2008). The Charlson comorbidity index is adapted to predict costs of chronic disease in primary care patients. J Clin Epidemiol.

[23] Saag LA, Tamhane AR, Batey DS, Mugavero MJ, Eaton EF (2018). Mental health service utilization is associated with retention in care among persons living with HIV at a university-affiliated HIV clinic. AIDS Res Ther.

[24] Centers for Disease Control and Prevention. Behavioral and Clinical Characteristics of Persons with Diagnosed HIV Infection. June 2019.

[25] Yehia BR, Stephens-Shield AJ, Momplaisir F, Taylor L, Gross R, Dube B (2015). Health outcomes of HIV-infected people with mental illness. AIDS Behav.

[26] Carrico AW, Bangsberg DR, Weiser SD, Chartier M, Dilworth SE, Riley ED (2011). Psychiatric correlates of HAART utilization and viral load among HIV-positive impoverished persons. AIDS (London, England).

[27] Bucek A, Leu CS, Benson S, Warne P, Abrams EJ, Elkington KS (2017). Psychiatric disorders, antiretroviral medication adherence, and viremia in a cohort of perinatally HIV-infected adolescents and young adults. Pediatric Infect Dis J.

[28] Leserman J (2008). Role of depression, stress, and trauma in HIV disease progression. Psychosom Med.

[29] Sin NL, DiMatteo MR (2014). Depression treatment enhances adherence to antiretroviral therapy: a meta-analysis. Ann Behav Med.

[30] Hoang T, Goetz MB, Yano EM, Rossman B, Anaya HD, Knapp H (2009). The impact of integrated HIV care on patient health outcomes. Med Care.

[31] Tater M PJ, Garfield R. Medi-Cal Managed Care: an overview and key issues. The Kaiser Commission on Medicaid and the Uninsured Issue Brief. March 2016.

[32] Arnold EA, Fuller S, Kirby V, Steward WT (2018). The impact of medicaid expansion on people living with HIV and seeking behavioral health services. Health Aff.

[33] Zuckerman S, Skopec L, M E. Medicaid Physician Fees after the ACA Primary Care Fee Bump. Washington, DC: Urban Institute, 2017 March.

