# Supplementary material for: The Impact of Mental Health Conditions on Public Insurance Costs of Treating HIV/AIDS
Source: AIDS Behav. 2019 Sep 6;24(6):1621–31. doi: 10.1007/s10461-019-02663-w (PMC7058503; doi:10.1007/s10461-019-02663-w)
Supplement: Supplementary file 1 — Electronic supplementary material 1 (DOCX 27 kb) [file 10461_2019_2663_MOESM1_ESM.docx]

Appendix

**Parameter estimates from regressions**

Medicare, full sample, outcome is logged total outpatient spending Medicaid, full sample, outcome is logged total outpatient spending

OLS regression OLS regression

Parameter Standard Parameter Standard

Estimate Error t Value Pr > |t| Estimate Error t Value Pr > |t|

Intercept 7.78276 0.02382 326.71 <.0001 Intercept 7.76433 0.02798 277.45 <.0001

Female gender 0.27730 0.03080 9.00 <.0001 Female gender 0.30262 0.02858 10.59 <.0001

African-American 0.10286 0.02592 3.97 <.0001 African-American 0.26161 0.03092 8.46 <.0001

Hispanic -0.11013 0.02514 -4.38 <.0001 Hispanic 0.05098 0.03580 1.42 0.1545

Other race/ethnicity -0.06473 0.04629 -1.40 0.1620 Other race/ethnicity -0.09975 0.04339 -2.30 0.0216

Age under 35 0.14265 0.05600 2.55 0.0109 Age under 35 -0.00611 0.04455 -0.14 0.8910

Age 50-64 0.07051 0.02106 3.35 0.0008 Age 50+ 0.16965 0.02691 6.30 <.0001

Age 65+ 0.19608 0.02988 6.56 <.0001 One comorbidity 0.45193 0.03007 15.03 <.0001

Dual Medi-Medi 0.35972 0.02124 16.94 <.0001 2+ comorbidities 1.14908 0.04250 27.04 <.0001

One comorbidity 0.64123 0.02218 28.91 <.0001 Mood disorders 0.42368 0.03465 12.23 <.0001

2+ comorbidities 1.44030 0.02840 50.71 <.0001 Adjustment/anxiety disorders 0.40864 0.05477 7.46 <.0001

Mood disorders 0.42473 0.02690 15.79 <.0001 Schizophrenia/other psychotic 0.45625 0.04884 9.34 <.0001

Adjustment/anxiety disorders 0.32789 0.04209 7.79 <.0001 One of the other 5 MH dx 0.48482 0.06166 7.86 <.0001

Schizophrenia/other psychotic 0.28233 0.04927 5.73 <.0001

One of the other 5 MH dx 0.20723 0.05521 3.75 0.0002

Medicare, full sample, outcome is having any inpatient stays/spending Medicaid, full sample, outcome is having any inpatient stays/spending

Logistic regression Logistic regression

Standard Wald Standard Wald

Estimate Error Chi-Square Pr > ChiSq Estimate Error Chi-Square Pr > ChiSq

Intercept -2.5462 0.0730 1217.4495 <.0001 Intercept -2.1096 0.0779 734.3187 <.0001

Female gender 0.1963 0.0792 6.1498 0.0131 Female gender 0.1489 0.0720 4.2720 0.0387

African-American 0.3068 0.0679 20.3844 <.0001 African-American 0.2429 0.0789 9.4797 0.0021

Hispanic 0.0885 0.0693 1.6301 0.2017 Hispanic -0.1148 0.0958 1.4357 0.2308

Other race/ethnicity -0.0965 0.1357 0.5052 0.4772 Other race/ethnicity -0.0588 0.1162 0.2560 0.6129

Age under 35 0.3615 0.1461 6.1252 0.0133 Age under 35 0.2798 0.1121 6.2276 0.0126

Age 50-64 -0.1013 0.0592 2.9296 0.0870 Age 50+ -0.1725 0.0704 6.0010 0.0143

Age 65+ -0.0444 0.0824 0.2900 0.5902 One comorbidity 1.1064 0.0730 229.8469 <.0001

Dual Medi-Medi 0.2487 0.0613 16.4788 <.0001 2+ comorbidities 2.4852 0.0980 642.7787 <.0001

One comorbidity 0.9231 0.0609 229.7085 <.0001 Mood disorders 0.5017 0.0835 36.1121 <.0001

2+ comorbidities 2.1037 0.0676 969.0556 <.0001 Adjustment/anxiety disorders 0.7050 0.1256 31.4957 <.0001

Mood disorders 0.4698 0.0684 47.2037 <.0001 Schizophrenia/other psychotic 0.7370 0.1114 43.7669 <.0001

Adjustment/anxiety disorders 0.1241 0.1070 1.3438 0.2464 One of the other 5 MH dx 0.5303 0.1415 14.0372 0.0002

Schizophrenia/other psychotic 1.3526 0.1106 149.5332 <.0001

One of the other 5 MH dx 0.3876 0.1297 8.9350 0.0028

Medicare, sample limited to those with any inpatient stays/spending Medicaid, sample limited to those with any inpatient stays/spending

Outcome is logged total inpatient spending Outcome is logged total inpatient spending

OLS regression OLS regression

Parameter Standard Parameter Standard

Estimate Error t Value Pr > |t| Estimate Error t Value Pr > |t|

Intercept 9.22132 0.08859 104.09 <.0001 Intercept 8.86796 0.07376 120.23 <.0001

Female gender -0.02239 0.08679 -0.26 0.7964 Female gender -0.11187 0.06294 -1.78 0.0757

African-American -0.01798 0.07552 -0.24 0.8118 African-American 0.05448 0.06943 0.78 0.4327

Hispanic 0.08588 0.08028 1.07 0.2849 Hispanic 0.06224 0.08743 0.71 0.4767

Other race/ethnicity 0.00887 0.16236 0.05 0.9564 Other race/ethnicity 0.18975 0.10645 1.78 0.0749

Age under 35 0.00263 0.16492 0.02 0.9873 Age under 35 0.28793 0.10098 2.85 0.0044

Age 50-64 -0.01908 0.06849 -0.28 0.7806 Age 50+ -0.14432 0.06248 -2.31 0.0210

Age 65+ -0.04884 0.09485 -0.51 0.6067 One comorbidity 0.24213 0.06966 3.48 0.0005

Dual Medi-Medi -0.16968 0.07200 -2.36 0.0185 2+ comorbidities 0.97479 0.07399 13.18 <.0001

One comorbidity 0.49881 0.07495 6.66 <.0001 Mood disorders -0.16030 0.07259 -2.21 0.0274

2+ comorbidities 1.19072 0.07461 15.96 <.0001 Adjustment/anxiety disorders 0.09377 0.09807 0.96 0.3392

Mood disorders -0.03393 0.07689 -0.44 0.6591 Schizophrenia/other psychotic 0.06981 0.09040 0.77 0.4401

Adjustment/anxiety disorders 0.33463 0.11241 2.98 0.0029 One of the other 5 MH dx 0.29701 0.10623 2.80 0.0052

Schizophrenia/other psychotic 0.21150 0.10507 2.01 0.0442

One of the other 5 MH dx 0.41100 0.12584 3.27 0.0011

Medicare, full sample, outcome is logged non-mental health outpatient spending Medicaid, full sample, outcome is logged non-mental health outpatient spending

OLS regression OLS regression

Parameter Standard Parameter Standard

Estimate Error t Value Pr > |t| Estimate Error t Value Pr > |t|

Intercept 7.76360 0.02392 324.52 <.0001 Intercept 7.73050 0.02809 275.17 <.0001

Female gender 0.28266 0.03093 9.14 <.0001 Female gender 0.31490 0.02869 10.98 <.0001

African-American 0.10352 0.02603 3.98 <.0001 African-American 0.27795 0.03104 8.95 <.0001

Hispanic -0.10666 0.02525 -4.22 <.0001 Hispanic 0.07353 0.03594 2.05 0.0408

Other race/ethnicity -0.05856 0.04649 -1.26 0.2078 Other race/ethnicity -0.11155 0.04356 -2.56 0.0105

Age under 35 0.14180 0.05624 2.52 0.0117 Age under 35 -0.05103 0.04473 -1.14 0.2539

Age 50-64 0.07610 0.02115 3.60 0.0003 Age 50+ 0.17761 0.02701 6.57 <.0001

Age 65+ 0.20526 0.03001 6.84 <.0001 One comorbidity 0.47368 0.03018 15.69 <.0001

Dual Medi-Medi 0.37364 0.02133 17.52 <.0001 2+ comorbidities 1.23922 0.04266 29.05 <.0001

One comorbidity 0.64433 0.02227 28.93 <.0001 Mood disorders 0.20203 0.03479 5.81 <.0001

2+ comorbidities 1.45188 0.02852 50.90 <.0001 Adjustment/anxiety disorders 0.25924 0.05498 4.71 <.0001

Mood disorders 0.32569 0.02702 12.05 <.0001 Schizophrenia/other psychotic -0.01134 0.04903 -0.23 0.8171

Adjustment/anxiety disorders 0.27414 0.04227 6.49 <.0001 One of the other 5 MH dx 0.13436 0.06190 2.17 0.0300

Schizophrenia/other psychotic 0.14708 0.04948 2.97 0.0030

One of the other 5 MH dx 0.19123 0.05545 3.45 0.0006

Medicare, full sample, outcome is having any non-mental health inpatient stays/spending Medicaid, full sample, outcome is having any non-mental health inpatient

Logistic regression stays/spending

Logistic regression

Standard Wald Standard Wald

Estimate Error Chi-Square Pr > ChiSq Estimate Error Chi-Square Pr > ChiSq

Intercept -2.5837 0.0742 1211.6259 <.0001 Intercept -2.0865 0.0784 709.1654 <.0001

Female gender 0.1885 0.0799 5.5745 0.0182 Female gender 0.1527 0.0726 4.4215 0.0355

African-American 0.2905 0.0686 17.9361 <.0001 African-American 0.2253 0.0796 8.0115 0.0046

Hispanic 0.0952 0.0701 1.8459 0.1743 Hispanic -0.1087 0.0970 1.2555 0.2625

Other race/ethnicity -0.1050 0.1383 0.5760 0.4479 Other race/ethnicity -0.0449 0.1178 0.1454 0.7029

Age under 35 0.3328 0.1501 4.9155 0.0266 Age under 35 0.2496 0.1143 4.7692 0.0290

Age 50-64 -0.0652 0.0600 1.1800 0.2774 Age 50+ -0.1559 0.0711 4.8112 0.0283

Age 65+ 0.0074 0.0827 0.0080 0.9286 One comorbidity 1.1372 0.0739 236.8845 <.0001

Dual Medi-Medi 0.2694 0.0621 18.8329 <.0001 2+ comorbidities 2.5397 0.0969 686.8201 <.0001

One comorbidity 0.9556 0.0620 237.7117 <.0001 Mood disorders 0.2131 0.0873 5.9550 0.0147

2+ comorbidities 2.1412 0.0677 1001.4905 <.0001 Adjustment/anxiety disorders 0.4345 0.1295 11.2573 0.0008

Mood disorders 0.2246 0.0716 9.8365 0.0017 Schizophrenia/other psychotic 0.0797 0.1195 0.4446 0.5049

Adjustment/anxiety disorders 0.1247 0.1091 1.3046 0.2534 One of the other 5 MH dx 0.2990 0.1456 4.2154 0.0401

Schizophrenia/other psychotic 0.3613 0.1198 9.0991 0.0026

One of the other 5 MH dx 0.3561 0.1311 7.3836 0.0066

Medicare, sample limited to those with any non-mental health inpatient stays/spending Medicaid, sample limited to those with any non-mental health inpatient stays/spending

Outcome is logged non-mental health inpatient spending Outcome is logged non-mental health inpatient spending

OLS regression OLS regression

Parameter Standard Parameter Standard

Estimate Error t Value Pr > |t| Estimate Error t Value Pr > |t|

Intercept 9.25400 0.09310 99.40 <.0001 Intercept 8.95368 0.07684 116.52 <.0001

Female gender -0.04006 0.09171 -0.44 0.6623 Female gender -0.09964 0.06605 -1.51 0.1316

African-American -0.02476 0.08005 -0.31 0.7572 African-American 0.00979 0.07329 0.13 0.8937

Hispanic 0.06442 0.08409 0.77 0.4437 Hispanic 0.01836 0.09174 0.20 0.8414

Other race/ethnicity -0.00151 0.17119 -0.01 0.9930 Other race/ethnicity 0.13114 0.11203 1.17 0.2420

Age under 35 -0.00204 0.18086 -0.01 0.9910 Age under 35 0.35595 0.10799 3.30 0.0010

Age 50-64 0.00151 0.07235 0.02 0.9833 Age 50+ -0.14427 0.06532 -2.21 0.0274

Age 65+ -0.03166 0.09834 -0.32 0.7475 One comorbidity 0.20695 0.07395 2.80 0.0052

Dual Medi-Medi -0.15815 0.07548 -2.10 0.0363 2+ comorbidities 0.89172 0.07752 11.50 <.0001

One comorbidity 0.45206 0.07989 5.66 <.0001 Mood disorders -0.12257 0.07821 -1.57 0.1173

2+ comorbidities 1.14380 0.07877 14.52 <.0001 Adjustment/anxiety disorders -0.11362 0.11044 -1.03 0.3037

Mood disorders 0.00119 0.08405 0.01 0.9887 Schizophrenia/other psychotic -0.01901 0.10481 -0.18 0.8561

Adjustment/anxiety disorders 0.21629 0.12504 1.73 0.0838 One of the other 5 MH dx 0.25505 0.12042 2.12 0.0343

Schizophrenia/other psychotic 0.13630 0.13320 1.02 0.3063

One of the other 5 MH dx 0.29735 0.13993 2.13 0.0337
